# Facilitators and Barriers to National COVID 19 Guideline Adherence among Healthcare Providers in Ethiopia

**DOI:** 10.4314/ejhs.v33i2.2

**Published:** 2023-03

**Authors:** Menbeu Sultan, Woldesenbet Waganew, Manuel Kassaye, Lemlem Beza, Miraf Waleligh, Aklilu Azazh, Sisay Yifru, Berhane Redae, Demelash Ataro, Asrat Fisseha, Aschalew Ashagre, Ararso Baru

**Affiliations:** 1 Saint Paul's hospital millennium medical college; 2 Jhpiego, Johns Hopkins; 3 Addis ababa Univercity; 4 Ministry of health Ethiopia; 5 Hawassa Univercity Hospital; 6 Arbaminch University

**Keywords:** Adherence, barriers, facilitators

## Abstract

**Background:**

Evidence-base practice needs to be supported by guidelines and decision-making protocols. This study aimed to look into the barriers and facilitators of adherence to national protocols in Ethiopia.

**Methods:**

Exploratory qualitative method was implemented to explore adherence to protocol. The national COVID-19 case management guideline was used as this study's prototype reference. A total of five FGDS were conducted among 26 healthcare providers. A total of 14 physicians and 12 nurses participated in the FGDs. Semi-structured focus group discussions guides were used to facilitate the discussion among healthcare workers involved in COVID-19 case management. The FGDs were audio recorded, transcribed and analyzed thematically.

**Results:**

Three broad themes have emerged from the content analysis. These include individual factors, environmental factors and system factors. System factors barriers to utilization include unclear guidelines, discordant guidelines and a lack of live national guidelines, while the main facilitator was supportive management. The environmental factors that were barriers to adherence included limited infrastructure and shortages of drugs suggested in the protocols.

**Conclusion:**

Outdated and discordant guidelines and a shortage of suggested managements were barriers. Future similar works should consider the identified barriers and need regular updates to facilitate effective implementation.

## Introduction

Practice guidelines and protocols serve as useful tools for clinical decision-making. Protocols are used to reduce practice variation, guide appropriateness and measure the quality of care (1). Ultimately, the goal of a guideline is to improve patient outcomes through the change to evidence-based practices. Unfortunately, substantial gaps have been documented between the development and dissemination of consensus statements and their implementation in practice (2). Adherence to practice guidelines is frequently used as a measure of the quality of care(2) (3). Valid and meaningful conclusions regarding physician adherence and its link to disease control rest on completing two tasks. First, physician adherence to the exciting guidelines must be assessed accurately. Second, the relation between physician adherence and outcome must be empirically demonstrated. Complete adherence to early goal-directed therapy has shown a significant reduction in the 28-day mortality rate, whereas partial adherence has not shown a beneficial effect(1) (4). It has been said low-quality health systems do as much harm, if not more, as a lack of access to universal health care and emphasize the expansion of access to universal health coverage accompanied by investments into the high-quality health system(5).

In Ethiopia, since the first diagnosis of COVID-19, there has been development and revision of the COVID-19 management protocol. The first national protocol was published in 2020 (6). It was developed with the involvement of consultants from different discipline and evaluation by societies(6). Studies have evaluated physician adherence to guidelines using prescription data, physician survey data, or medical record reviews. And it has been reported that implementation of the guidelines in resource-limited settings is very challenging (7) (8). But research on barriers and facilitators to protocol adherence among healthcare providers, especially communicable disease, are limited in Ethiopia. Therefore, this study aimed to look into the barriers and facilitators of adherence to national protocols among healthcare providers in Ethiopia.

## Methods

**Study design and setting**: An exploratory qualitative research method was implemented to explore healthcare workers adherence to national COVID-19 case management guideline. To address the study objectives, participants were recruited from four COVID-19 treatment centers found in Ethiopia. The participating COVID-19 centers were involved in the implementation and promotion of adherence to the national COVID-19 clinical management guidelines. All the selected centers are the largest and the first treatment centers dedicated for COVID 19 in Ethiopia. They had at least 200 dedicated beds for COVID 19 treatment with critical care service including mechanical ventilation. They had implemented at least two versions of the national COVID-19 management guidelines.

Based on the aforementioned precondition for inclusion, we recruited the study participants from St Paul Millennium Medical College, Eeka Kotebe General Hospital, Millennium Hall COVID-19 Treatment Center, and Hiwot Fana Comprehensive Specialized Hospital COVID-19 Treatment Center. The first three centers were in Addis Ababa while Hiwot Fana Comprehensive Specialized Hospital COVID-19 Treatment Center was in Harar City Administration, Eastern Ethiopia.

**Data collection procedures**: Focus group discussions were conducted among selected healthcare workers using semi-structured FGD guides. The discussions were held with doctors and nurses working at the selected COVID-19 treatment centers. Onsite discussions were held with the participants recruited from Addis Ababa while online (webinar) discussion using Zoom was held with participants recruited from Hiwot Fana comprehensive specialized Hospital COVID-19 Treatment Center due to logistic issues. The focus group discussions were held on November 2021.

Each FGD was facilitated by PhD candidates who were experienced in research methodology. A total of 26 participants that comprised 14 physicians and 12 nurses were included in the study. Each FGD included providers involved in the clinical management of COVID-19 patients in intensive care units, emergency departments, inpatient care, and at least one person involved in leadership roles. The FGD was conducted until we reached thematic saturation, which was achieved on the 4^th^ FGD and we added one FGD to confirm the saturation further. Each discussion lasted 52 to 70 minutes. To manage dominance during a discussion, all participants were given a turn, and the pace was determined by the low-level professionals.

**Data analysis**: The discussions were audio recorded and have listened to repeatedly. The content was transcribed, organized under related themes, and verbatim indicated. Some participants who preferred to express their idea in the local language were allowed to do so and translated into English. An inductive thematic analysis approach was used because it provides a flexible, rich and detailed account of qualitative data. The transcribed verbatim was read several times, coded, grouped, categorized and abstracted the final themes. For both barriers and facilitator factors, three broad themes have emerged from the content analysis. The broad themes included health system factors, environmental factors, and individual factors. Our findings are further described in subthemes. Selected quotes were provided under identified themes.

**Data quality assurance**: The researchers involved in this study were familiar with the study context and the technical guideline. While interpreting the data, the effort was made to maintain the original meanings. Peer debriefing was also done among the study team during data analysis.

**Ethical consideration**: The ethical clearance was obtained from the Ethiopian public health institute's institutional review board (IRB) with the approval number EPHI-IRB-288-2020. Participation was voluntary, and there was no finical benefit for the research participants. The study objective and significance were explained to the study participants, and written informed consent was obtained from the participants before commencing FGDs.

## Results

As summarized in figure one below, three broad themes emerged from the content analysis. The broad themes included health system, environmental, and individual factors.

**Figure 1:**
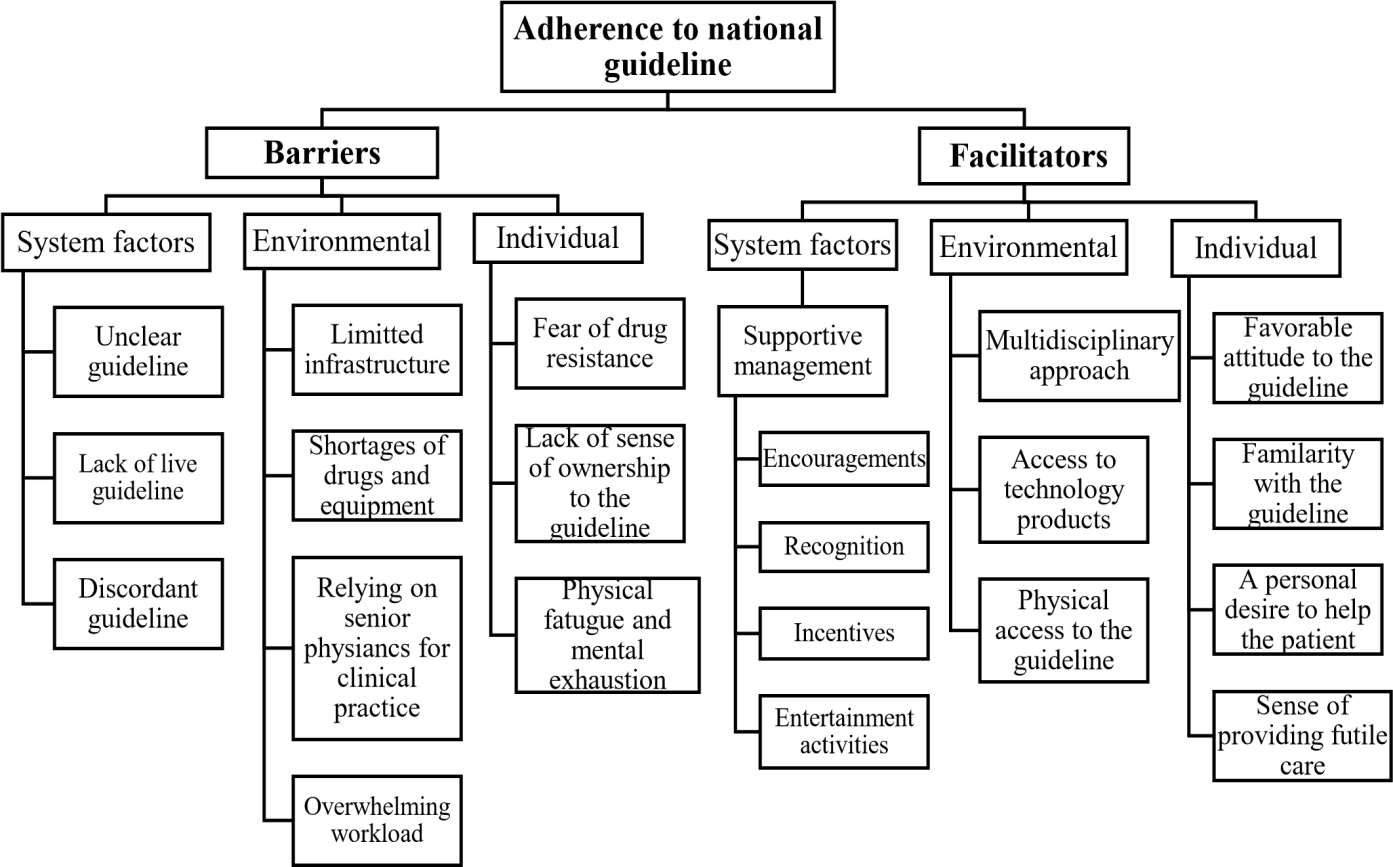
Facilitators and barriers of adherence to national COVID-19 management guideline in Ethiopia, 2021.

**Demography of participants**: A total of 26 participants were involved in five FGDs. Twelve participants were consultant doctors trained in internal medicine, and emergency medicine and critical care. Two of the participants were general doctors. There were five MSc nurses in emergency and critical care; the remaining seven were senior BSc nurses. All participants were working in the COVID-19 treatment center for at least six months prior to the study, and five of them were leaders of their centers. Thirteen (50%) of the FGD participants were female.

**1. System and Guideline Related Barriers to Protocol Adherence**

**Health system factors**: Unclear, discordant and lack of live (up to date) national guideline were the three sub themes identified as a barrier in this theme

**Unclear guideline**: Lack of clarity on some of the content of the guideline was observed to have an impact on adherence to the guideline. Physicians expressed difficulty in practicing general recommendations written on the guideline to specific patients. The following exemplifies lack of clarity and ambiguity in the national guideline.

*“The guideline is open ended and lack details. For example, duration for administering antibiotics was not specified on the protocol.”* (FGD3, P1)

*“Uncertainty in prescribing some drugs were common. For example, the guideline did not mention whether to administer prophylactic or therapeutic dose of heparin when there is indication to administer.”* (FGD 2, P3)

**Discordant guideline**: Discordance among the guidelines were noticed and created uncertainties in choosing appropriate guideline to practice.

*“Our hospital guideline recommends antibiotics for COVID-19 patients only if there is evidence of bacterial super-infection while the national guideline recommend antibiotics for all critically ill COVID-19 patients. Literatures and other international guidelines support our institution's guideline.”* (FGD1, P3)

**Lack of live (up to date) national guideline**: Given the fact that COVID-19 is a recent phenomenon, the local (institutional) guideline and the WHO guidelines were changing continuously. Meanwhile, delayed edition was noticed in the national guideline. As a result, the health care provider was forced to look for other updated guidelines. The participants lamented the situation as follows,

*“We have sedation protocol at hospital level, which is relatively updated compared to national protocol. Currently, we are implementing the local protocol for sedation”* (FGD1, P2).

**2. Environmental factors to Protocol Adherence**

**Limited infrastructure**: Lack of adequate space to isolate and treat COVID-19 patients was identified as a barrier to the implementation of COVID-19 national guideline. In addition, lack of accommodation at the center for healthcare providers treating COVID-19 patients and shortage of transportation services hampered their adherence to the guideline. The following quotes typified the sentiments of the study participants:

*“Our hospital's infrastructure was not initially designed to accommodate COVID-19 patients. We converted some of the blocks at our hospital to COVID-19 treatment center. The rooms were not adequately ventilated and the spaces were very limited to isolate the patients. We used to admit COVID-19 patients in corridor and open spaces during the hit time although it was against the recommendation.”* (FGD3, P5).

*“We had many patients who could have been benefited if admitted to intensive care unit but kept in ward due to limited access to infrastructure. We gave priorities to salvageable patients due to lack of resources. Lack of adequate infrastructures were hindering our adherence to the guideline.”* (FGD2, P6) *transport. The situation is worst during the night*

**Shortages of medicines and equipment mentioned on the guideline**: Shortage of oxygen caused preventable deaths and other adverse patient outcomes. Inability to provide care due to shortage of oxygen and other supplies brought job dissatisfaction of care providers. One of the participants narrated his devastating experience as follow:

*“I cannot forget a postpartum woman, whom we lost likely due to oxygen hunger. She gave birth through cesarean section and sent to our center after testing positive for the virus. She was on mechanical ventilator and weaned off after improvement... she was, then, on minimum oxygen. Sadly, the hospital run out of oxygen and we lost the patient after all those efforts. It was the worst experience we had in treating COVID-19 patients” (FGD3, P1).*

**Overwhelming workload**: The respondents mentioned that they were overwhelmed with workload at the centers during the crisis peak and they perceived that it affected their adherence to the guideline.

*“It was the toughest moment in my career. At the beginning, the center implemented one nurse for one patient. But later the approach changed due to the shortage of manpower. I was providing care to several patients during the same shift. Lack of attendants worsen the situation. Personal protective equipment was additional burden.” (FGD 2, P2).*

**3. Individual factors to Protocol Adherence**

**Fear of drug resistance**: Healthcare workers' fear of antibiotic resistance made them less adherent to the national COVID-19 management guideline, as it recommends potent antibiotics for all critically ill COVID-19 patients. This reserved many physicians participating in this study from implementing the guideline according to the recommendations.

*“I am worried that antibiotics resistance is inevitable if we continue to stick to the guideline (national) and prescribe potent antibiotics for all critically ill COVID-19 patients... Personally, I prescribe antibiotics only when there is evidence of bacterial superinfection.”* (FGD2, P4)

**Job stress and burnout**: Several discussants reported adherence to the national guideline could be varied as time goes on (decision fatigue). They added that the difference in practice could also exist between the day and night shifts. Most of the participant agreed that the difference in adherence to the guideline could be due to mental depletion as the time went on and physical fatigue due to the overwhelming workload.

*“The clinicians' adherence to the guideline and performance may decrease during the night shift due to lack of adequate sleep. But I don't think the difference is significant and could affect the patient outcome.”* (FGD2, P5)

*“The pandemic is not giving us break although there was a periodic rise and fall of the cases. We are continuously dealing with it for the last 18 months. We are burnout. We need rest from the pandemic.”* (FGD3, P3)

**Sense of providing futile care**: Some respondent mentioned that perceived sense of futile care prevented them from taking some care indicated on the guideline.

*“Intubating COVID-19 patients could be considered as palliative care as we experienced less chance of survival among intubated patients. Sometimes, we delay intubation against the indication mentioned on the guideline to benefit the patient.”* (FGD1, P3).

**Facilitators of Protocol Utilization**

**System factors**

**Supportive management**

Our findings explored that adherence to the COVID-19 national protocol was influenced by the level of support provided to health care providers. Supportive management promoted adherence to the national COVID-19 management protocol. Incentives, encouragement, recognitions and entertainment activities enhanced adherence to the COVID-19 protocol. One of the participants described the management situation as follow:

*“The hospital management was very supportive to us. There was continuous encouragement from the management team. They organized events to entertain us. We have attractive incentives. Their words of encouragement personally motivated me to practice according the protocol.”* FGD 2, P3.

*“We have good communication with the management. They listen to us. We have attractive incentives, which very is important for staff's motivation and adherence to the protocol.”* FGD 1, P4.

On the other hand, less supportive management negatively impacted protocol adherence. One of the study participants stated that

*“We didn't have incentive payment for the last five months. Most of our staffs are currently less motivated to work...No doubt! Delayed payment and false reassurance from the management is affecting quality of the care and our compliance with the guideline*.” FGD4, P3.

**Environmental factors**

**Multidisciplinary team approach in the implementation of the guideline**: The guideline encourages collaborative work and multidisciplinary approach in the patient management, which enhanced adherence to the guideline. Some of the respondent lamented the experience as follow:

*“Multidisciplinary approach enhanced our adherence to the guideline. The center had access to pulmonologists, hematologists, intensivists, cardiologists, and many others. They work together on patients' management.”* FGD2, P1.

*“Our center uses multidisciplinary approach to manage COVID-19 patients. Each professional has responsibility to implement the guideline and I hope it helped us.”* FGD 5, P2.

**Senior physicians influence**: The discussants mentioned that senior physicians were one of the main trusted sources of the most updated knowledge on COVID-19. Some of the participants claimed that they changed their practice based on the information they heard from senior physicians to benefit the patients. The participant believed that some of the senior ‘physicians’ recommendations may deviate from the guideline or not present in the guideline. However, they strongly trust them and include their recommendations in their practice, believing they have access to the most updated evidence.

*“We have senior physicians, who are readers and continuously updating their knowledge with the growing evidences on COVID-19. Sometimes, we depend on their recommendation to guide some of our daily practices, particularly for the recommendation that lack clarity on the national guideline.”* FGD2, P5.

*“Our hospital was the pioneering COVID-19 center in Ethiopia. Many of our senior physicians participated in the preparation of the national guideline. We have access to those physicians and sometimes depend on their recommendation for some actions that we perceive outdated on the national guideline”* FGD2, P1

**Access to technology**: The study participants mentioned that access to technology such as smart phone, internet and telegram application enhanced adherence to COVID-19 guideline.

*“I had access to smart phone and internet at workplace. We have also telegram group for our center. We share soft copy of the document through telegram. It enhanced availability of the guideline to us.”* FGD 4, P3.

**Physical access to the guideline**: Physical accessibility of the guideline to health care workers was reported as one of the facilitators of the guideline adherence. The following quote explains one of the discussant experiences.

*“I have the soft copy version of the guideline in my phone. Printed version of the document is also available at the workplace. Some recommendations from the guideline were also posted on the wall of the patient room. We usually refer to it as a guidance.”* FGD4, P2

**Individual factors**

**Familiarity with the guideline**

All the participants were familiar with at least two versions of the national COVID-19 management guidelines. The participants acknowledged that familiarity with the guideline facilitated their adherence to the protocol.

*“I am familiar with the national COVID-19 management protocol including the third edition (which is the most recent version). I am also familiar with the update made to each version. I have soft copy of the document in my cellphone and refer to it when needed even at the patient's bedside.”* FGD 4, P4

*“I know the guideline from the beginning. It was comprehensive and touched all aspects of the patients. If you are familiar with the content of the guideline, you would likely implement it.” FGD 5, P5.*

**Attitude toward the guideline**: Health care providers good attitude toward the guideline enhanced adherence to the guideline. All the respondents reported that the national guideline is essential document for their clinical practices. One of the respondents explained his attitude towards the national COVID-19 management guideline as follow:

*“The guideline was developed based on evidences generated from researches and other international guidelines. Therefore, it has great contribution for the care we provide to our patients. We should adhere to the guideline to achieve the minimum recommended standard of care.”* FGD1, P3

**Sense of ownership to the guideline**: The participant reported that involving their own institution or someone they know in their institution in the guideline development by valuing their contribution, creates a sense of ownership to the guideline and enhance adherence. Similarly, when healthcare workers institution is not involved in the guideline development process, less sense of ownership and adherence to the guideline were reported.

*“Our center had great input for the national guideline. Our senior staffs contributed to its development. I can say the guideline reflects our hospital and our activity is also in line with the guideline.”* FGD2, P4

*“We had anesthesiologists, emergency medicine specialists, internists, and other highly trained professionals. However, none of them were invited to the preparation of the guideline. If they were involved in the preparation, we could have more comprehensive guideline with higher sense of ownership.”* FGD 4, P2.

**A compassionate care to help the patient**: Health care providers personal desire to help their patients and the satisfaction they got from improving patients were reported as facilitators of adherence to the guideline. The following are some of the quotes taken from the FGD,

*“Although what I am getting and the workload is not matching, I believe that it is my duty to adhere to the guideline and provide better care to my patient. Seeing patients recover was source of my personal satisfaction.”* FGD4, P2.

## Discussion

This study explored facilitators and barriers of adherence to national COVID-19 management guidelines in Ethiopia. This study revealed healthcare providers adherence to national guidelines is influenced by individual, environmental and system related factors. Being unfamiliar with guideline was among the key factors that impede physicians adherence to guideline (7,8). Those providers who were familiar with the updated guideline likely implement the guideline. On the other hand, this study identified that healthcare workers familiarity with the guideline facilitate adherence to COVID-19 guideline.

Adherence to the guideline could be affected by the nature of the guideline itself. Adherence to the guideline could be affected by the nature of the guideline itself. Previous studies identified Unclear guideline as one of the leading barriers in implementing guideline (9–11). In this study, lack of information clarity was identified as one of the barriers in adhering to the national COVID-19 management guideline. The guideline should include concrete recommendations how information be applied to be effectively adhered (10). A clear and easy to understand guideline has a greater chance of being used (12).

It was reported that when the applicability of the guideline to clinical practice is questionable, the providers utilize the guideline less (8). Similarly, we have identified several scenarios supporting this argument in this study. As shown in this study, the participants perceived a sense of futile care when there was no progress in the patient's condition after implementing the recommendations have disparaged the use of the guideline. We also noted that many of the study participants cited concerns about fear of drug resistance if they fully adhered to the guideline, which recommends the prescription of empiric antibiotics for all critically ill COVID-19 patients without observing evidence of bacterial superinfection. It has been previously described that if physicians perceived the guideline was insufficiently evidence-based, they were less likely to adhere to it (7).

Poor adherence to the national guideline was also noted when there is a mismatch in the recommendations between the hospitals and the national guideline. The participants described that, healthcare providers were more adherent to the local guideline despite the availability of a national guideline in their hospital mentioning that some of the information recommended by national guideline were outdated. Healthcare providers reported strict adherence to guideline if they felt their institution or someone they know was involved in the development of the guideline (8,15). A guideline is more likely to be successful if the end users are involved in the development of the guideline. This can be achieved through their societies and by inviting stakeholders to comment before the launching of the guideline (16).

The role of supportive management in influencing adherence to clinical guideline was well established in previous literatures (12,17,18). This study also found that feeling supported by the management was seen to influence healthcare workers adherence to the national COVID-19 guideline. Providers who received incentives and psychological supports reported strict adherence to COVID-19 management guideline. It was also reported that mandating the guidelines implementation with a multi-disciplinary team enhances adherence to a guideline (14), which is in agreement with our study findings.

Having access to internet and smart phone helped the healthcare provider adherence to the guideline. Other studies also revealed, access to smart phone significantly increases clinician adherence to guideline (19). The present study also found that the use of social media platform such as telegram group for information dissemination facilitated provider adherence to the COVID-19 guideline. As healthcare workers are usually busy with their work, the use of multi-faceted approach to communication could enhance their access to information thereby facilitate their adherence to guideline (10).

Additionally, previous studies have reported that adequate infrastructure, drug and medical equipment supply are necessary for achieving full adherence to clinical guidelines (20–22). Similarly, inadequate space to isolate and treat COVID-19 patients, lack of access to equipment recommended on the guideline (for example, ABG machine), and scarcity of drugs including oxygen were amongst the barriers that prevented the participants from full adherence to the guideline in the present study.

The present study has several limitations. First, participant's recruitment to the study was limited to COVID-19 treatment centers found in Addis Ababa and Harar city administration. This could limit generalizability of the findings to the entire nation. Secondly although we did not notice any difference in data collection between virtual meeting and physical FGD,FGD conducted with participants recruited from Hiwot Fana comprehensive specialized hospital COVID-19 treatment center was done via webinar. Therefore, the non-verbal expressions were not fully observed and recorded for all participants. Though the study uses a qualitative approach which has its own limitations to generalize, quantitative estimates are required to identify determinants of guideline adherence.

This study identified the major system, environmental and individual factors that influenced care providers adherence to national COVID-19 case management guideline. To improve patient outcomes and promote evidence-based practice, the major factors such as Unclear guideline, discordant guideline and lack of live (up to date) national guideline should be considered. In addition infrastructure and availability of drugs should be considered during guide line development.

## Data Availability

All the data included in the manuscript can be accessed from the corresponding author Menbeu Sultan Mohammed upon request through an email address of smenbeu@tahoo.com

## References

[1] Quintero RA, Martínez CA, Gamba JD, Ortiz I (2012). Adherence to international guidelines on early management in severe sepsis and septic shock. Biomedica.

[2] Almazrou SH, Alfaifi SI, Alfaifi SH, Hakami LE, Al-Aqeel SA (2020). Barriers to and Facilitators of Adherence to Clinical Practice Guidelines in the Middle East and North Africa Region: A *Systematic Review*. Healthcare.

[3] Cavazos JM, Naik AD, Woofter A, Abraham NS (2008). Barriers to physician adherence to nonsteroidal anti-inflammatory drug guidelines: A qualitative study. Aliment Pharmacol Ther.

[4] Corley A, Hammond NE, Fraser JF (2010). The experiences of health care workers employed in an Australian intensive care unit during the H1N1 Influenza pandemic of 2009: A phenomenological study. Int J Nurs Stud.

[5] Vogel JP, Moore JE, Timmings C, Khan S, Khan DN, Defar A (2016). Barriers, Facilitators and Priorities for Implementation of WHO Maternal and Perinatal Health Guidelines in Four Lower-Income Countries: A GREAT Network Research Activity. PLoS ONE.

[6] https://covidlawlab.org/wp-content/upload/2020/06/National-Comprehensive-COVID19-Management-Handbook.pdf.

[7] Cavazos JM, Naik AD, Woofter A, Abraham NS (2008). Barriers to physician adherence to nonsteroidal anti-inflammatory drug guidelines: A qualitative study. Aliment Pharmacol Ther.

[8] Forsner T, Hansson J, Brommels M, Wistedt AT, Forsell Y (2010). Implementing clinical guidelines in psychiatry: A qualitative study of perceived facilitators and barriers. BMC Psychiatry.

[9] Corley A, Hammond NE, Fraser JF (2010). The experiences of health care workers employed in an Australian intensive care unit during the H1N1 Influenza pandemic of 2009: A phenomenological study. Int J Nurs Stud.

[10] Locatelli SM, Lavela SL, Hogan TP, Kerr AN, Weaver FM (2012). Communication and information sharing at VA facilities during the 2009 novel H1N1 influenza pandemic. Am J Infect Control [Internet].

[11] Chau JPC, Thompson DR, Twinn S, Lee DTF, Lopez V, Ho LSY (2008). An evaluation of SARS and droplet infection control practices in acute and rehabilitation hospitals in Hong Kong. Hong Kong Med J.

[12] Francke AL, Smit MC, De Veer AJE, Mistiaen P (2008). Factors influencing the implementation of clinical guidelines for health care professionals: A systematic meta-review. BMC Med Inform Decis Mak.

[13] Kang JH, Kim EJ, Choi JH, Hong HK, Han SH, Choi IS (2018). Difficulties in using personal protective equipment: Training experiences with the 2015 outbreak of Middle East respiratory syndrome in Korea. Am J Infect Control [Internet].

[14] Almazrou Mazrou S (2013). Expected benefits of clinical practice guidelines: Factors affecting their adherence and methods of implementation and dissemination. J Heal Spec.

[15] Feyissa GT, Woldie M, Munn Z, Lockwood C (2019). Exploration of facilitators and barriers to the implementation of a guideline to reduce HIV-related stigma and discrimination in the Ethiopian healthcare settings: A descriptive qualitative study. PLoS One.

[16] Verschueren KJC, Kodan LR, Brinkman TK, Paidin RR, Henar SS, Kanhai HHH (2019). Bottom-up development of national obstetric guidelines in middle-income country Suriname. BMC Health Serv Res.

[17] Saillour-Glenisson F, Michel P (2003). Facteurs individuels et collectifs associés à l'application des recommandations de pratique clinique par le corps médical. Revue de la littérature. Rev Epidemiol Sante Publique.

[18] Woith W, Volchenkov G, Larson J (2012). Barriers and motivators affecting tuberculosis infection control practices of Russian health care workers. Int J Tuberc Lung Dis.

[19] Yoon CH, Ritchie SR, Duffy EJ, Thomas MG, McBride S, Read K (2019). Impact of a smartphone app on prescriber adherence to antibiotic guidelines in adult patients with community acquired pneumonia or urinary tract infections. PLoS One.

[20] Wong ELY, Wong SYS, Lee N, Cheung A, Griffiths S (2012). Healthcare workers' duty concerns of working in the isolation ward during the novel H1N1 pandemic. J Clin Nurs.

[21] Akshaya KM, Shewade HD, Aslesh OP, Nagaraja SB, Nirgude AS, Singarajipura A (2017). “Who has to do it at the end of the day? Programme officials or hospital authorities?” Airborne infection control at drug resistant tuberculosis (DR-TB) centres of Karnataka, India: A mixed-methods study. Antimicrob Resist Infect Control.

[22] Buregyeya E, Nuwaha F, Verver S, Criel B, Colebunders R, Wanyenze R (2013). Implementation of tuberculosis infection control in health facilities in Mukono and Wakiso districts, Uganda. BMC Infect Dis.

